# Flow benefits of pediatric arterial cannulae versus introducer sheaths as distal perfusion catheters in venoarterial extracorporeal membrane oxygenation

**DOI:** 10.1016/j.xjon.2025.09.023

**Published:** 2025-09-24

**Authors:** Joshua G. Crane, Mark S. Slaughter, Steven C. Koenig, Gretel Monreal

**Affiliations:** a Department of Cardiovascular and Thoracic Surgery, University of Louisville, Louisville, Ky; b Department of Bioengineering, University of Louisville, Louisville, Ky

**Keywords:** venoarterial extracorporeal membrane oxygenation, VA-ECMO, arterial cannula, introducer sheath, distal perfusion catheter, DPC, mock flow loop, limb ischemia

## Abstract

**Objective:**

We test the hypothesis that pediatric arterial (“peds art”) cannulae as distal perfusion catheters (DPCs) for venoarterial extracorporeal membrane oxygenation provide more favorable distal limb hemodynamics than introducer sheaths.

**Methods:**

Introducer sheaths (Teleflex 5, 6, 8 Fr) and peds art cannulae (Medtronic 6, 8, 10 Fr) were tested as DPCs in static and dynamic mock loops. Flow through each and flow loss caused by their intravascular obstructiveness was measured to calculate pressure gradient versus flow, intravascular obstruction flow, and resistances.

**Results:**

All 3 peds art cannulae tested delivered greater flows than the 3 introducer sheaths. The 10-Fr peds art cannula provided the most flow (0.97 L/min, 1500 rpm), whereas all 3 introducer sheaths provided much lower and nearly identical flows (only 0.18-0.20 L/min, 1500 rpm) despite their different diameters. The 10-Fr peds art cannula provided the most flow at equivalent head pressures despite being the most obstructive (least amount of flow around it, 2.0 L/min) relative to no DPC (2.5 L/min), resulting in the highest cumulative flow distally.

**Conclusions:**

The 10-Fr peds art cannula was the most beneficial DPC tested. All peds art cannulae tested possessed a better tradeoff of flow delivery versus intravascular obstructiveness compared to introducer sheaths, resulting in more favorable distal limb hemodynamics. The integrated stopcock with small holes (0.061′′ = 4.65 Fr) on the introducer sheath's sidearm and at the 90° sidearm's attachment to the sheath's hub increase resistance. These chokepoints of introducer sheaths prevent sufficient flow to support distal limb perfusion as DPC.

**Figure:**
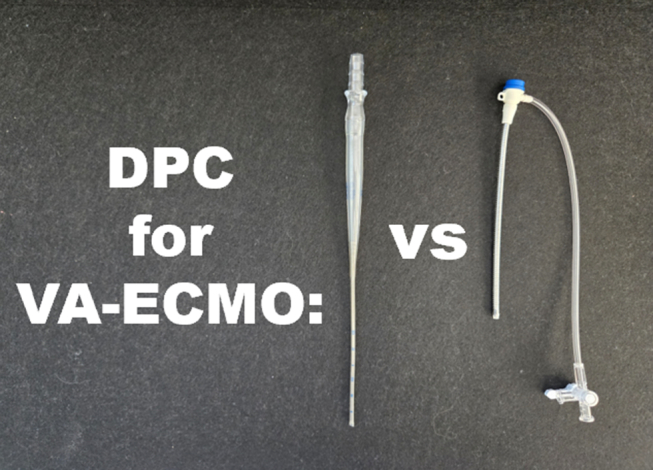
We investigate pediatric arterial cannulae versus introducer sheaths as DPC in VA-ECMO.

**Central Message**

Pediatric arterial cannulae as distal perfusion catheters for VA-ECMO may result in more favorable distal limb hemodynamics compared with introducer sheaths.

**Perspective**

Introducer sheaths are often used as distal perfusion catheters (DPCs) to improve lower extremity blood flow and prevent limb ischemia in VA-ECMO; however, clinical outcomes remain mixed. We test the hypothesis that pediatric arterial cannulae as DPCs provide more favorable distal limb hemodynamics than introducer sheaths.

Limb ischemia is the most frequent vascular complication in patients who receive venoarterial extracorporeal membrane oxygenation (VA-ECMO), with the reported incidence ranging from 10% to 70%.^1^, ^2^, ^3^, ^4^, ^5^, ^6^, ^7^, ^8^ To improve blood flow distal to the femoral artery cannula and/or resolve/reduce the risk of limb ischemia, a distal perfusion catheter (DPC) is often used per Extracorporeal Life Support Organization recommendations.^9^ A small-caliber introducer sheath (5- to 8-Fr diameter) is commonly used as the DPC,^10^, ^11^, ^12^, ^13^, ^14^ integrated into the ECMO circuit via its sidearm using the Luer lock port on the arterial cannula and placed distal to the femoral artery cannula. Although DPCs are used both prophylactically and reactively, clinical outcomes (mortality, leg ischemia) remain mixed.^1^^,^^3^^,^^6^^,^^15^^,^^16^

Using a dynamic mock flow loop model of adult femoral VA-ECMO, we previously showed that increasing the size (French) of the introducer sheath as a DPC did not improve distal limb hemodynamics, and that there was a tradeoff between flow through the introducer sheath and the degree of its intravascular obstructiveness.^1^ Introducer sheaths are designed for percutaneous delivery of intravascular catheters/tools via the hemostatic valve and for pressure monitoring, blood sampling, and the administration of various agents (ex. fluids, contrast, etc) via the sidearm, not necessarily for providing blood flow to support distal limb perfusion. We speculate that the integrated stopcock on the introducer sheath's sidearm and the 90° sidearm port attachment to the sheath's hub are chokepoints for delivering sufficient DPC flow. We hypothesize that using a cannula specifically designed for delivering blood flow, such as a pediatric arterial cannula, may result in more favorable distal limb hemodynamics over using an introducer sheath. Using static and dynamic flow loop models as the experimental platform, we investigated the hydrodynamics and hemodynamics of pediatric arterial cannulae versus introducer sheaths as DPC in femoral VA-ECMO.

## Methods

**Figure 1:**
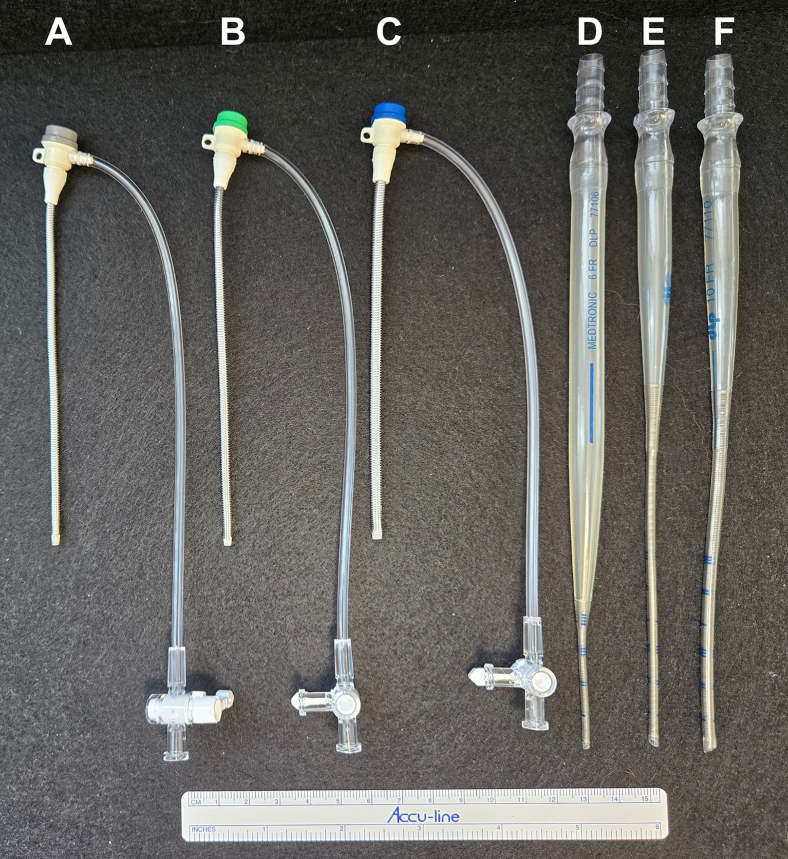
Photo of the 3 introducer sheaths and 3 pediatric arterial cannulae tested as distal perfusion catheters in this study. A, 5-Fr Super Arrow-Flex Percutaneous Sheath Introducer, 11 cm, CP-07511 (Arrow/Teleflex). B, 6-Fr Super Arrow-Flex Percutaneous Sheath Introducer, 11 cm, CP-07611 (Arrow/Teleflex). C, 8-Fr Super Arrow-Flex Percutaneous Sheath Introducer, 11 cm, CP-07811 (Arrow/Teleflex). D, 6-Fr Pediatric arterial cannula, 22.9 cm, 77106 (Medtronic). E, 8-Fr Pediatric arterial cannula, 22.9 cm, 77108 (Medtronic). F, 10-Fr Pediatric arterial cannula, 22.9 cm, 77110 (Medtronic).

No institutional review board or patient consent was required for this in vitro study. Static and dynamic mock flow loop experiments were performed as described previously^1^^,^^2^^,^^17^^,^^18^ to evaluate the hydrodynamic and hemodynamic performance of 3 different introducer sheaths and 3 different pediatric arterial cannulae as DPC for VA-ECMO (Figure 1):

1. 5-Fr Super Arrow-Flex Percutaneous Sheath Introducer, 11 cm, CP-07511 (Arrow/Teleflex).
2. 6-Fr Super Arrow-Flex Percutaneous Sheath Introducer, 11 cm, CP-07611 (Arrow/Teleflex).
3. 8-Fr Super Arrow-Flex Percutaneous Sheath Introducer, 11 cm, CP-07811 (Arrow/Teleflex).
4. 6-Fr Pediatric arterial cannula, 22.9 cm, 77106 (Medtronic).
5. 8-Fr Pediatric arterial cannula, 22.9 cm, 77108 (Medtronic).
6. 10-Fr Pediatric arterial cannula, 22.9 cm, 77110 (Medtronic).

The 5-Fr, 6-Fr, and 8-Fr introducers sheaths (Figure 1) each had an integrated stopcock (0.061″ inner diameter hole) and a sidearm (0.061″ inner diameter hole) with a 90° bend connecting the sidearm (0.074″ inner diameter) to the wire-wrapped introducer sheath, as measured using a set of calibrated pin gauges (Vermont Gage). The stopcock and sidearm hole sizes (0.061″ diameter = 4.65 Fr) were the same for each introducer sheath, regardless of sheath diameter (French size).

### Static Mock Flow Loop – DPC (in Series Placement) Flow Experiments

**Figure 2:**
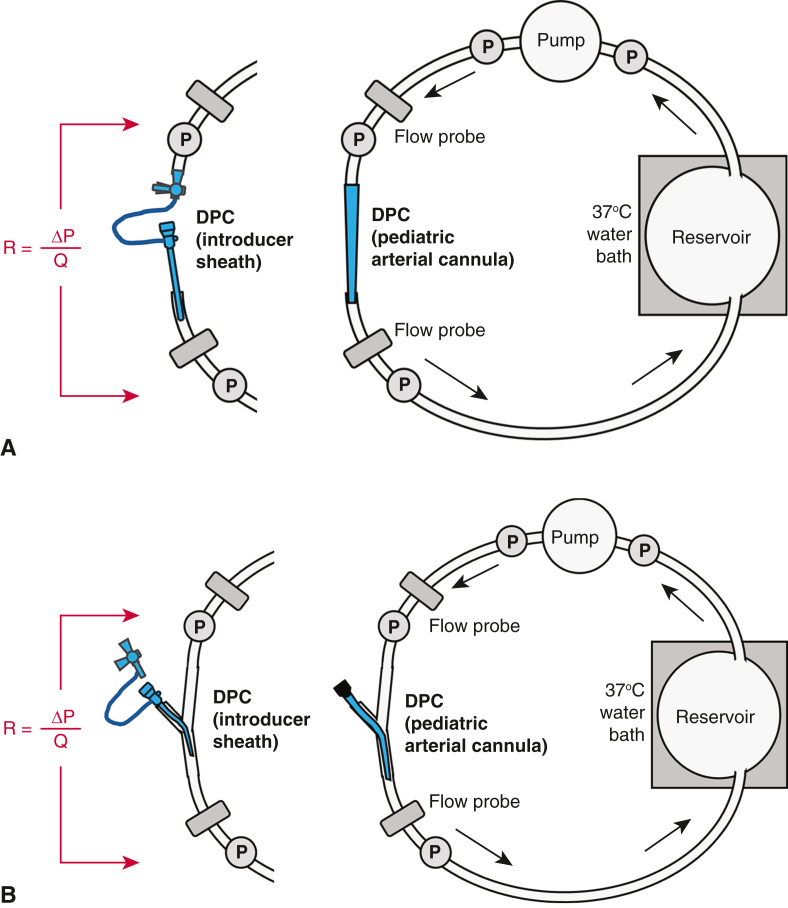
A, Schematic of the static mock flow loop used for the in-series flow experiments. Each of the 6 distal perfusion catheters (*DPCs*) tested were integrated serially (in-line) into the loop so that all pump flow passed through the DPC only (no other venoarterial extracorporeal membrane oxygenation (*VA-ECMO*) components such as the arterial and venous cannulae and membrane oxygenator). P = the location of each pressure transducer. Flow resistance (*R*) of each DPC was calculated as the pressure gradient (*ΔP*) across the DPC divided by flow (Q) through the DPC. B, Schematic of the static mock flow loop used for the flow obstructiveness experiments. Each of the 6 DPCs tested were integrated into the loop via a Y-connector with Tuohy and closed/capped so that all pump flow passed around the DPC (no flow through DPC). Obstructive resistance (*R*) of each DPC was calculated as the pressure gradient (*ΔP*) across the DPC divided by flow (Q) around the DPC (no flow through the DPC).

Static mock flow loop experiments were performed to assess each DPC's hydrodynamic performance independent of other ECMO components (no arterial and venous cannulae, no membrane oxygenator). Each DPC was selected in random order and integrated serially (in-line with pump flow) into the static mock loop circuit (Tygon 3350) as shown in Figure 2, *A*, with all of the loop (pump) flow passing through the DPC. The loop was primed with 660 mL of 3.4cP glycerol-distilled water solution at 37 °C. Viscosity was validated using Cannon-Fenske calibrated viscometers (ACE Glass) following ASTM Standard D445-21E2. The loop's reservoir was kept in a water bath to maintain the circuit at 37 °C. A centrifugal pump (Affinity; Medtronic) with driver (73-5157; Harvard Apparatus) was used to provide flow through the circuit. Four fluid-filled transducers (MLT0380/D; ADInstruments) were calibrated using a manometer (Meriam) and used to acquire pressure data at DPC inlet and outlet and pump inlet and outlet positions. Transit-time flow probes (6PXL with TS410 modules; Transonic Systems) were used to measure flow proximal and distal to the DPC (pre- and post-DPC). Data were acquired from 500-1500-500 rpm in 100-rpm pump increments (resulting in 2 sets of data) at a 200/s sampling rate using a PowerLab 16/35 acquisition system with bridge amps (ADInstruments) and recorded using LabChart v.8.1.30 (ADInstruments). Resistance (R) of each DPC was calculated as Δ pressure across the DPC divided by the flow through the DPC. Data were plotted using Prism, version 10.5.0 (GraphPad).

### Static Mock Flow Loop – DPC (Intravascular Placement) Flow Obstructiveness Experiments

To assess “intravascular obstructiveness,” each DPC was integrated into the static mock loop circuit via a Y-connector with Tuohy and then closed/capped so that all loop flow passed around the DPC (no flow through the DPC) (Figure 2, *B*). These experiments were performed as described previously.

### Dynamic Mock Flow Loop

**Figure 3:**
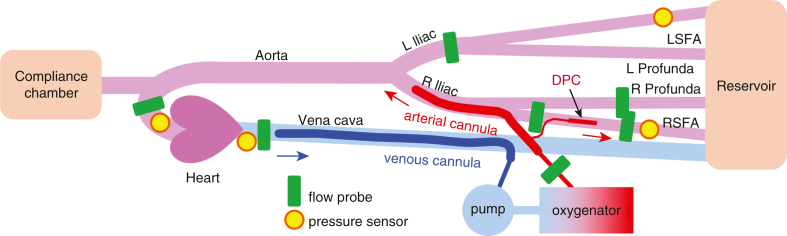
Schematic of the dynamic mock flow loop model of adult femoral venoarterial extracorporeal membrane oxygenation (*VA-ECMO*) used for investigating the hemodynamics of distal perfusion catheters (*DPCs*). Each of the 6 DPCs (*red*) tested were integrated into the right superficial femoral artery (*RSFA*) of the loop. The arterial cannula (*red*, 15-Fr) was placed in the right common femoral artery and the venous cannula (*blue*, 25-Fr) was placed in vena cava. Pressures (*yellow*) were measured at the aorta (*Ao*), RSFA, left superficial femoral artery (*LSFA*), and vena cava positions. Flows (*green*) were measured at the Ao, RSFA pre-DPC, RSFA post-DPC, right profunda femoral artery, left iliac artery, vena cava, and ECMO pump outlet.

A dynamic loop was constructed (Figure 3) to mimic adult femoral VA-ECMO. Tubing was selected to replicate average adult vessel diameters while working within the constraints of standard tubing and flow probe sizes (Tygon 3350; 3/4″ for the aorta, 3/8″ for the iliac arteries, 1/4″ for the femoral arteries, and 3/4″ for the vena cava).^19^ The silicone ventricle (Derby City Supply) was powered by a pneumatic driver (Ventricular Assist Device Pneumatic Drive System, Thoratec [now Abbott]) that controlled ventricular filling, ejection, and beat rate. Silicone valves (Minivalve International) were placed in the aortic and mitral positions. An arterial cannula (15-Fr CB96570-015, 18 cm; Medtronic) and venous cannula (25-Fr 96600-125, 76.2 cm; Medtronic) were integrated into the loop via Tuohy connectors (Medical Murray). A centrifugal pump (Affinity; Medtronic) and driver (73-5157; Harvard Apparatus) served as the ECMO pump. A membrane oxygenator (US5062; Eurosets) was integrated into the loop.

Fluid-filled transducers (MLT0380/D; ADInstruments) were calibrated using a manometer (Meriam) and used to acquire pressure data at the aortic, right superficial femoral artery (RSFA), left superficial femoral artery, and vena cava positions. Transit-time flow probes (PXL with TS410 modules; Transonic Systems) were placed on the aorta, RSFA pre-DPC, RSFA post-DPC, right profunda femoral artery, left iliac artery, ECMO outflow, and vena cava positions. The dynamic loop was primed with 3.6 L of 3.4cP glycerol-distilled water solution at 37 °C. Viscosity was validated using Cannon-Fenske calibrated viscometers (ACE Glass) following ASTM Standard D445-21E2. The reservoir was kept in a temperature-controlled water bath to maintain the circuit at 37 °C. The dynamic loop was tuned to heart failure conditions with a heart rate of 80 bpm, mean aortic flow of 3.0 ± 0.5 L/min, mean aortic pressure of 50 ± 5 mm Hg, and central venous pressure of 20 ± 5 mm Hg.

The same 6 DPCs described for the static mock loop experiments were integrated into the dynamic loop. Each DPC was connected to the arterial cannula and integrated into the loop's RSFA. Experiments were performed with no DPC integrated into the loop, with the DPC integrated into the loop but clamped off from the arterial cannula (no flow through the DPC), and with the DPC integrated into the loop and patent. Data were recorded at pump speeds of 0, 1000, 2000, and 3000 rpm for each of the 6 DPCs tested and then again decrementally (resulting in 2 sets of data), and collected at a 200/s sampling rate using a PowerLab 16/35 acquisition system with bridge amps (ADInstruments) and recorded using LabChart v.8.1.30 (ADInstruments). Data were plotted using Prism, version 10.5.0 (GraphPad).

## Results

### Static Mock Flow Loops

**Figure 4:**
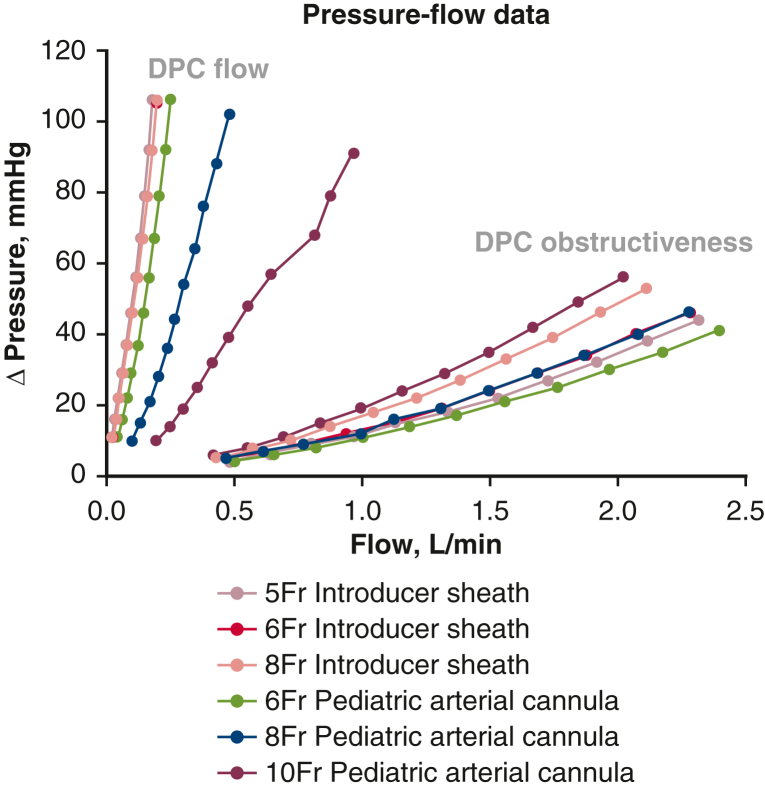
Results from the static mock flow loop experiments showing pressure gradient (*ΔP*) versus flow across each of the 6 distal perfusion catheters (*DPCs*) tested with flow delivered by a centrifugal pump from 500 to 1500 rpm, 100 rpm increments. “DPC flow” is the flow through each DPC tested; “DPC obstructiveness” is the flow within the mock loop tubing around each DPC tested (no flow through the DPC itself). Note these experiments were performed in duplicate but only one set of data is presented on the graph for clarity purposes.

Experiments were performed to quantify the hydrodynamics of the 6 DPCs. Pressures and flows were measured with the pump flow passing through each DPC only (in series configuration) to characterize pressure gradient-flow (ΔP-Q) curves and to calculate DPC resistance (R). Flow through the DPC was the highest in the 10-Fr pediatric arterial cannula, followed by the 8-Fr and then 6-Fr pediatric arterial cannulae, respectively (Figure 4). The 10-Fr pediatric arterial cannula was able to provide 0.97 L/min flow at a pump speed of 1500 rpm. Flow through the DPC was the lowest in the 5-Fr introducer sheath (0.18 L/min flow at 1500 rpm) and the 6-Fr and 8-Fr introducer sheaths only provided 6.2% and 10.1% more flow, respectively; thus, the 3 introducer sheaths provided much lower and nearly identical flow, regardless of diameter (French size).

Average DPC pressure gradient and flow were calculated at steady-steady state for each pump speed with the mock loop pump flow passing through each DPC (as a measure of DPC flow in series configuration) and then around each DPC (as a measure of each DPC's “intravascular” obstructiveness) as shown in Figure 4. The 10-Fr pediatric arterial cannula provided the most flow at equivalent head pressures (ΔP) while also being the most obstructive, resulting in the least amount of flow around the DPC (2.0 L/min, 1500 rpm) relative to no DPC in the static loop (2.5 L/min, 1500 rpm). Conversely, the 6-Fr pediatric arterial cannula and the 5-Fr introducer sheath were the least obstructive while also delivering the least amount of DPC flow.

**Figure 5:**
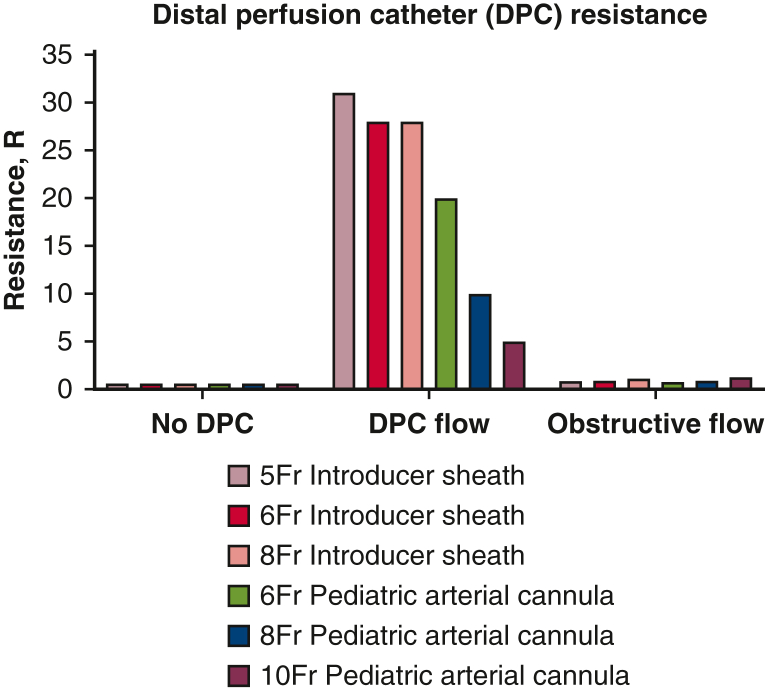
Calculated resistances for each of the 6 distal perfusion catheters (*DPCs*) tested in the static mock flow loop for no DPC (*left*), DPC in series with pump (*center*), and DPC obstructiveness (*right*) experimental configurations. Resistance (*R*) of each DPC was calculated as the pressure gradient (*ΔP*) across the DPC divided by flow (Q) through the DPC (*center*) or around the DPC (*right*).

The DPC flow resistances were much greater than the obstructive flow resistances for all DPC tested and there were minimal differences in obstructive resistances between the 6 DPC tested and compared to no DPC resistances (Figure 5). These findings demonstrate DPC size (5-10 Fr) has minimal impact on intravascular flow (obstructiveness) and are primarily dependent on DPC flow resistance.

### Dynamic Mock Flow Loops

**Figure 6:**
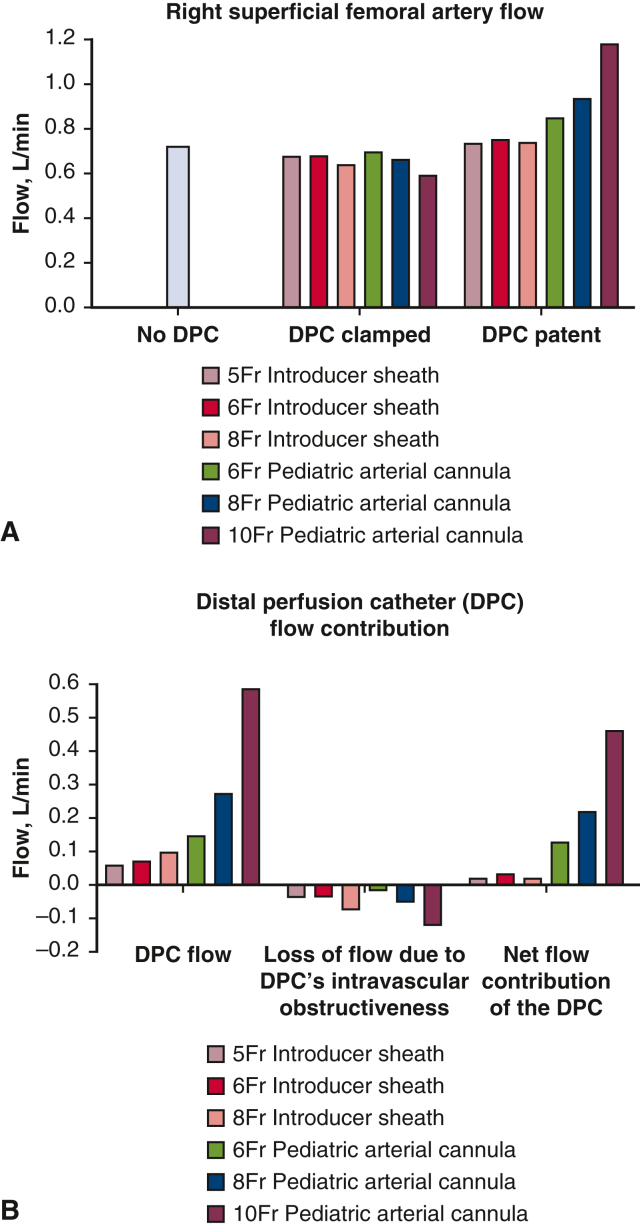
Right superficial femoral artery (*RSFA*) and distal perfusion catheter (*DPC*) flow data from the dynamic mock flow loop at 3000 rpm pump speed. A, RSFA (the DPC-cannulated artery) data showing the flow effects of no DPC in the RSFA, the 6 different DPC each placed in the RSFA but clamped (no arterial cannula flow through the DPC), and the 6 different DPCs each placed in the RSFA and patent (receiving flow from the arterial cannula). B, calculated flow contributions from each of the six DPC tested showing forward flow through each DPC (*left*), flow loss attributable to DPC “intravascular” obstructiveness (*middle*), and the net DPC flow (= DPC flow + DPC obstructed flow) added cumulatively (= RSFA flow + net DPC flow) to the RSFA (*right*).

**Figure 7:**
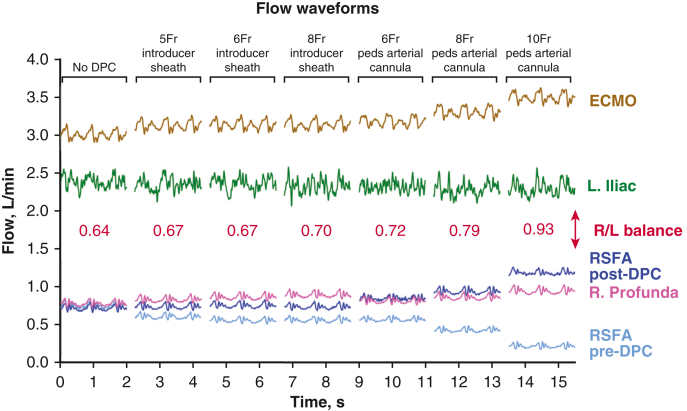
Flow waveforms recorded from the dynamic mock loop during venoarterial extracorporeal membrane oxygenation (*VA-ECMO*) support at a pump speed of 3000 rpm with no distal perfusion catheter (*DPC*) in the circuit and with the 6 different DPCs each placed in the right superficial femoral artery (*RSFA*) and patent (receiving flow from the arterial cannula). Flow data measured from the RSFA pre- and post-DPC, the right profunda (R. profunda), left iliac artery (L. iliac), and from the ECMO pump. Show in *red* are the right and left leg flow balances (R/L balance) for each DPC tested (right leg flow = RSFA post-DPC flow + R. profunda flow; left leg flow = L. iliac flow).

Experiments were performed using a dynamic mock flow loop model of adult femoral VA-ECMO to quantify the hemodynamic effects of the 6 different DPCs tested. Each DPC was integrated into the loop's RSFA, as shown in Figure 3. Experiments were performed with no DPC integrated into the loop, with the DPC integrated into the loop but clamped off from the arterial cannula (no flow through the DPC), and with the DPC integrated into the loop and patent (DPC receiving flow from the arterial cannula). Each of the 3 pediatric arterial cannulae provided more flow to the RSFA than with no DPC in the ECMO circuit, whereas all 3 of the introducer sheaths provided equal (or less) flow to the RSFA, despite being similarly sized (6 Fr, 8 Fr) DPCs (Figure 6, *A*). Specifically, the 10-Fr pediatric arterial cannula provided the highest DPC flow (Figure 6, *B*, *left*) and overcame the largest obstructive flow (Figure 6, *B*, *middle*) to deliver the greatest net forward flow through the RSFA (Figure 6, *B*, *right*). The 6-Fr and 8-Fr pediatric arterial cannulae also delivered increases of >100 mL/min added to the cumulative RSFA flow, whereas in contrast each of the 3 introducer sheaths delivered minimal net flow increases to cumulative RSFA flow (= RSFA flow + DPC net flow, where DPC net flow = DPC flow + DPC obstructed flow). ECMO flow, post-RSFA flow (= RSFA + net DPC flow). Right profunda flow increased and pre-RSFA flow decreased with increasing sized introducer sheaths and pediatric arterial cannulae with VA-ECMO at 3000 rpm compared to VA-ECMO with no DPC, with the pediatric arterial cannulae having greater increasing/decreasing responses compared to each of the 3 introducer sheaths, as shown in Figure 7.

Other observations from our static and dynamic mock flow loop experiments included increased ECMO flow with increasing DPC diameter independent of pump speed (rpm), with the greatest ECMO flow achieved with use of the 10-Fr pediatric arterial cannula as DPC (Figure 7). There were minimal differences in aortic or vena cava flow for any of the 6 DPCs tested at each of the ECMO pump speeds in the dynamic loop (data not shown). Each of the 3 pediatric arterial cannulae increased post-RSFA and right profunda femoral artery flow, decreased pre-RSFA flow, and had no effect on left iliac artery flow. The 10-Fr pediatric arterial cannula achieved the best right leg (post-RSFA flow + right profunda flow)/left leg (left iliac flow) flow balance of 0.93, whereas the 5-Fr and 6-Fr introducer sheaths (0.67 for both) only slightly improved the right/left leg flow balance compared with no DPC (0.64) (Figure 7).

## Discussion

Use of a DPC is considered standard of care in VA-ECMO to minimize the risk of limb ischemia: the Extracorporeal Life Support Organization recommends placement of a DPC within 4 hours of femoral artery cannulation.^9^ DPCs are used both prophylactically and reactively, yet clinical outcomes (mortality, leg ischemia) remain mixed.^1^^,^^3^^,^^6^^,^^15^^,^^16^ Introducer sheaths are frequently used as DPC^10^, ^11^, ^12^, ^13^, ^14^ and are integrated into the ECMO circuit via their sidearm using the Luer lock port on the arterial cannula and placed distal to the femoral artery cannula. We previously investigated the potential hemodynamic benefits, limitations, and/or tradeoffs as a function of arterial cannula size, use and size of DPCs, and pump speed (ECMO flow) during VA-ECMO in a dynamic flow loop.^1^ We found that a larger arterial cannula size resulted in decreased flow, pressure, and pulsatility in the distal femoral artery and its branches, with contrasting increased flow and pressure in the non-cannulated femoral artery. These divergent effects (increased flow in the contralateral leg) were independent of whether no DPC, a 5-Fr introducer sheath DPC, or an 8-Fr introducer sheath DPC was used.^1^ We also found that increasing the DPC size did not result in measurable hemodynamic benefits (dynamic mock loop study),^1^ an observation also reported by Zipfel and colleagues^14^ in a retrospective clinical study. In this study, we specifically investigated the hemodynamic benefits and limitations of introducer sheaths as DPC during VA-ECMO support and compared to use of pediatric arterial cannulae as a potential alternative DPC.

Mohite and colleagues^11^ performed a retrospective study examining patients with VA-ECMO at their institution who had received an introducer sheath versus a pediatric arterial cannula as the DPC and found that use of a pediatric cannula was associated with a lower incidence of limb ischemia. Simons and colleagues^10^ also reported using 9-Fr or 11-Fr pediatric aortic cannulae (Medtronic) as DPC, and like Mohite and colleagues,^11^ noted the advantages of the larger inner lumen, laminar flow, avoidance of the 90° sidearm with introducer sheaths that may increase turbulence and shear, and advantages of a direct connection to the arterial cannula's tubing without the use of a stopcock.^10^^,^^11^ Foltan and colleagues^20^ recognized the introducer sheath's integrated stopcock limits DPC flow and modified their sidearm with larger tubing and no stopcock.

Introducer sheaths are designed for percutaneous delivery of intravascular catheters/tools via the hemostatic valve and for pressure monitoring, blood sampling, and the administration of various agents (eg, fluids, contrast, etc) through the sidearm. They are not necessarily designed to deliver blood flow to support distal limb perfusion. Many introducer sheaths have an integrated stopcock, and the stopcock hole is quite small relative to the sheath's inner diameter. The 5-Fr, 6-Fr, and 8-Fr introducer sheaths that we used in this study have been used clinically as DPC at our institution and have integrated stopcocks on their sidearms. We surmised that the integrated stopcock on the introducer sheath's sidearm and the small 90° sidearm attachment to the sheath hub may be chokepoints restricting/limiting flow, which may be insufficient to provide distal limb perfusion as DPC. Each of the stopcock holes and the sidearm port holes measured 0.061” (as measured using a set of pin gauges). These hole sizes, the equivalent of 4.65 Fr, were the same for each introducer sheath tested, regardless of sheath size, which may help explain why introducer sheath flow were nearly identical regardless of sheath size (Figure 4). In addition, these restrictions (holes) in the introducer sheaths may also increase shear and the potential risk for blood damage (eg, hemolysis), which may warrant further investigation.

In addition to the higher flows delivered to the RSFA, the pediatric arterial cannulae tested, which can be placed open or percutaneously, provided more favorable hemodynamic benefits compared to the introducer sheaths: (1) lower DPC resistance (Figure 5) and (2) better right/left leg flow balance (Figure 7). The resistance in the RSFA due to DPC intravascular obstructiveness was much smaller (minimal) compared with the flow through the DPC for both introducer sheaths and pediatric cannulae independent of their diameters. Each of the 6 DPCs tested were placed in parallel with the ECMO arterial cannula, thereby lowering total resistance which led to increased ECMO and RSFA (right leg) flows. In addition, because of their much lower DPC resistances, the pediatric cannulae delivered greater flows and better right/left leg flow balances compared with all 3 introducer sheaths. We also observed only minimal differences in aortic and vena cava flow regardless of DPC size and type. This indicates that rather than flow being shunted down the leg (ie, pulling from the VA-ECMO flow), the overall VA-ECMO flow is increased thereby providing adequate central perfusion and distal limb perfusion.

### Limitations

We only tested one brand of introducer sheath and one brand of pediatric arterial cannulae as DPC; it is possible other introducers and cannulae would result in different hemodynamic data. The 6-Fr pediatric arterial cannula tested had a different taper than the 8-Fr and 10-Fr versions (Figure 1), despite being part of the same Medtronic DLP One-Piece Pediatric Arterial Cannula line of products. We performed our experiments using a glycerol solution instead of blood so we cannot quantify the potential risk for shear or thrombosis. Our dynamic mock loop is a lumped parameter model designed to simulate the average adult human vasculature in heart failure. We did not investigate different patient and/or vessel sizes in our mock loop models, as this was beyond the scope of this study. Our static and dynamic loops simulate hemodynamics, mechanical properties (resistance, compliance, inertance), and fluid viscosity, but are unable to replicate the microvasculature, physiological neurohormonal response(s), and vascular injury as seen in the human body.

In summary, the pediatric arterial cannulae tested as DPC possess the better tradeoff of flow delivery versus intravascular obstructiveness over the introducer sheaths tested, resulting in much more favorable distal limb hemodynamics during VA-ECMO. The 10-Fr pediatric arterial cannula achieved the highest flow despite the largest obstructiveness, providing the most flow to the RSFA due to much lower resistance (to flow through the DPC). This is the first study to experimentally investigate the potential hemodynamic benefits of pediatric arterial cannulae as DPC compared with introducer sheaths in VA-ECMO. Our findings demonstrate hemodynamic advantages of using pediatric arterial cannulae as DPC and suggest potential clinical benefits in preventing and/or resolving limb ischemia during VA-ECMO, which should next be validated in a small clinical study.

## Conflict of Interest Statement

Drs Monreal, Koenig, and Slaughter are investigators on National Institutes of Health (NIH) grant R44HL144214 unrelated to this project. Dr Monreal is an investigator on a grant from Abiomed unrelated to this project. Dr Koenig is an investigator on NIH grant R01HL150346 unrelated to this project. Dr Slaughter is on the advisory board of Medtronic. Dr Crane is supported by the Mansbach Research Endowment Fund. Dr Monreal has received honoraria from the European Science Foundation and is supported in part by a gift from Robert M. Prizant to the Legacy Foundation of Kentuckiana.

The *Journal* policy requires editors and reviewers to disclose conflicts of interest and to decline handling or reviewing manuscripts for which they may have a conflict of interest. The editors and reviewers of this article have no conflicts of interest.

## References

[1] Crane J.G., Monreal G., Koenig S.C., Slaughter M.S. (2025). Hemodynamic considerations of distal perfusion catheters with venoarterial extracorporeal membrane oxygenation: a dynamic mock loop study. ASAIO J.

[2] Crane J.G., Monreal G., Koenig S.C., Slaughter M.S. (Published online June 27, 2025). Hemodynamic considerations of ipsilateral vs contralateral cannulation with venoarterial extracorporeal membrane oxygenation. J Thorac Cardiovasc Surg Open.

[3] Nejim B., Snow R., Chau M. (2025). Acute limb ischemia in patients on veno-arterial extracorporeal membrane oxygenation (VA-ECMO) support: a ten-year single-center experience. Ann Vasc Surg.

[4] Hart J.P., Davies M.G. (2024). Vascular complications in extracorporeal membrane oxygenation—a narrative review. J Clin Med.

[5] Bonicolini E., Martucci G., Simons J. (2019). Limb ischemia in peripheral veno-arterial extracorporeal membrane oxygenation: a narrative review of incidence, prevention, monitoring, and treatment. Crit Care.

[6] Elmously A., Bobka T., Khin S. (2018). Distal perfusion cannulation and limb complications in venoarterial extracorporeal membrane oxygenation. J Extra Corpor Technol.

[7] Yang F., Hou D., Wang J. (2018). Vascular complications in adult postcardiotomy cardiogenic shock patients receiving venoarterial extracorporeal membrane oxygenation. Ann Intensive Care.

[8] Gouchoe D.A., Chaurasia S., Henn M.C. (2024). Does size matter? The effect of size of distal perfusion catheter on acute limb ischemia: a meta-analysis. ASAIO J.

[9] Richardson A.S.C., Tonna J.E., Nanjayya V. (2021). Extracorporeal cardiopulmonary resuscitation in adults. Interim guideline consensus statement from the Extracorporeal Life Support Organization. ASAIO J.

[10] Simons J., Di Mauro M., Mariani S. (2024). Bilateral femoral cannulation is associated with reduced severe limb ischemia-related complications compared with unilateral femoral cannulation in adult peripheral venoarterial extracorporeal membrane oxygenation: results from the Extracorporeal Life Support Registry. Crit Care Med.

[11] Mohite P.N., Fatullayev J., Maunz O. (2014). Distal limb perfusion: achilles' heel in peripheral venoarterial extracorporeal membrane oxygenation. Artif Organs.

[12] Zhou X., Chen B., Hu C. (2025). A tip for assessing blood flow in distal perfusion catheter during veno-arterial extracorporeal membrane oxygenation. Crit Care.

[13] Kaufeld T., Beckmann E., Ius F. (2019). Risk factors for critical limb ischemia in patients undergoing femoral cannulation for venoarterial extracorporeal membrane oxygenation: is distal limb perfusion a mandatory approach?. Perfusion.

[14] Zipfel S., Pecha S., Hakmi S. (2016). Distal limb perfusion—a necessary third cannula in extracorporeal membrane oxygenation. J Thorac Cardiovasc Surg.

[15] Juo Y.Y., Skancke M., Sanaiha Y., Mantha A., Jimenez J.C., Benharash P. (2017). Efficacy of distal perfusion cannulae in preventing limb ischemia during extracorporeal membrane oxygenation: a systematic review and meta-analysis. Artif Organs.

[16] Son A.Y., Khanh L.N., Joung H.S. (2021). Limb ischemia and bleeding in patients requiring venoarterial extracorporeal membrane oxygenation. J Vasc Surg.

[17] Monreal G., Koenig S.C., Slaughter M.S. (2022). Feasibility testing of the Inspired Therapeutics NeoMate mechanical circulatory support system for neonates and infants. PLoS One.

[18] Monreal G., Koenig S.C., Kelley J.F. (2024). Early-stage development of the CoRISMA mechanical circulatory support (CMCS) system for heart failure therapy. Cardiovasc Eng Technol.

[19] Pearce W.H., Slaughter M.S., LeMaire S. (1993). Aortic diameter as a function of age, gender, and body surface area. Surgery.

[20] Foltan M., Philipp A., Göbölös L. (2019). Quantitative assessment of peripheral limb perfusion using a modified distal arterial cannula in venoarterial ECMO settings. Perfusion.

